# Pilot Influenza Syndromic Surveillance System Based on Absenteeism and Temperature in China: Development and Usability Study

**DOI:** 10.2196/37177

**Published:** 2022-10-14

**Authors:** Zhen Yang, Chenghua Jiang

**Affiliations:** 1 School of Medicine Tongji University Shanghai China; 2 Dongfang Hospital Tongji University Shanghai China

**Keywords:** influenza, syndromic surveillance system, face recognition, infrared thermometer, absenteeism, temperature

## Abstract

**Background:**

Shortcomings of the current school-based infectious disease syndromic surveillance system (SSS) in China include relying on school physicians to collect data manually and ignoring the health information of students in attendance.

**Objective:**

This study aimed to design and implement an influenza SSS based on the absenteeism (collected by face recognition) and temperature of attending students (measured by thermal imaging).

**Methods:**

An SSS was implemented by extending the functionality of an existing application. The system was implemented in 2 primary schools and 1 junior high school in the Yangtze River Delta, with a total of 3535 students. The examination period was from March 1, 2021, to January 14, 2022, with 174 effective days. The daily and weekly absenteeism and fever rates reported by the system (DAR1 and DFR; WAR1 and WFR) were calculated. The daily and weekly absenteeism rates reported by school physicians (DAR2 and WAR2) and the weekly positive rate of influenza virus (WPRIV, released by the Chinese National Influenza Center) were used as standards to evaluate the quality of the data reported by the system.

**Results:**

Absenteeism reported by school physicians (completeness 86.7%) was 36.5% of that reported by this system (completeness 100%), and a significant positive correlation between them was detected (*r*=0.372, *P*=.002). When the influenza activity level was moderate, DAR1s were significantly positively correlated among schools (*r*_ab_=0.508, *P*=.004; *r*_bc_=0.427, *P*=.02; *r*_ac_=0.447, *P*=.01). During the influenza breakout, the gap of DAR1s widened. WAR1 peaked 2 weeks earlier in schools A and B than in school C. Variables significantly positively correlated with the WPRIV were the WAR1 and WAR2 of school A, WAR1 of school C, and WFR of school B. The correlation between the WAR1 and WPRIV was greater than that between the WAR2 and WPRIV in school A. Addition of the WFR to the WAR1 of school B increased the correlation between the WAR1 and WPRIV.

**Conclusions:**

Data demonstrated that absenteeism calculation based on face recognition was reliable, but the accuracy of the temperature recorded by the infrared thermometer should be enhanced. Compared with similar SSSs, this system has superior simplicity, cost-effectiveness, data quality, sensitivity, and timeliness.

## Introduction

School-aged children are vulnerable to influenza. The incidence of influenza among children aged 5-14 years during the influenza season ranges from 17.31% to 46.61% [1]. School-aged children also contribute to the amplification of virus transmission. Epidemiologic evidence suggests that influenza first occurs among school-aged children and that once infected, this population transmits the virus among family members and subsequently to the general population [2,3]. An infected student spreads the virus to an estimated 2.4 (95% CI 1.8-3.2) other children within a school [4]. Incidence rates in adults who reside with school-aged children may be 2 to 3 times higher than the incidence rates in similar adults who do not reside with school-aged children [5].

Absenteeism is an influenza surveillance indicator recommended by the World Health Organization and an important determinant adopted in the school-based syndromic surveillance system (SSS). In 1979, Peterson et al [6] demonstrated the effectiveness of school absenteeism for influenza surveillance. Since then, several studies have discussed the value of absenteeism surveillance from different perspectives. Compared to other methods, the main advantages of absenteeism surveillance include noninvasiveness, no requirement of clinical testing, low cost, simple operation, and good representation. Additionally, absenteeism surveillance can be used to accurately estimate the economic burden of influenza and its impact on education and promote effective cooperation between the health and education departments [6-12]. However, absenteeism itself is not a direct manifestation of the clinical symptoms of the disease, but only an approximate estimate of the illness. Therefore, it is sensitive but lacks specificity [10]. Baer et al [11] suggested that the greatest value of absenteeism surveillance may lie in “situational awareness” rather than “early detection.”

Absenteeism can be divided into three types: all-cause absence, illness absence, and syndrome-specific absence [13]. These 3 indicators have high specificity, but they increase the workload of the school. The SSS must balance specificity and the burden on the school [12]. Otherwise, the schools will be uncooperative, and the system will be eventually rejected [11,14,15]. Reducing the burden to maintain and improve the compliance of schools is an important challenge for the effective operation of such systems. Researchers have continuously been focusing on three main technical approaches to address this challenge: (1) improving the automation of data collection and changing the data collection method from manual statistics to fingerprint [14] or smart card methods [16]; (2) improving the convenience of data transmission, with data transmission changing from postcards, telephone, and fax [6] to email [10], and then to widely used network platforms [7-9,11,14-16]; (3) diversifying data reporters, with the reporter usually being a school physician or teacher and some systems requiring parents to report information [15,17] or encouraging students to participate in reporting the information [18].

Public health emergencies of infectious diseases emerging from schools account for 85.64% of the total number of all annual national public health emergencies [19]. Therefore, school-based SSSs are particularly significant in China. The SARS outbreak in 2003 and the H1N1 pandemic in 2009 prompted many places in mainland China to build systems for school-based infectious disease symptom surveillance [15,20-22], but the continuous operation of such systems is challenging. First, although the government formulated the school infectious disease reporting standard as early as 2012 [23] and issued several documents emphasizing the reporting of epidemic information in schools during the COVID-19 pandemic [24,25], absenteeism statistics are still not mandatory for schools. Second, the severe shortage of school physicians exacerbates the problem. Only 33.1% of primary and secondary schools are equipped with school physicians, with 1 school physician serving 2800 students on average; the proportions of school physicians in central and western regions, rural areas, and low-grade schools are even lower [26]. Therefore, the system based on manual information reports struggles to stimulate enthusiasm in schools [16]. Finally, Chinese people attach significant importance to studying, and parents worry that absence will delay the learning progress of their children. Consequently, they will send their children to school even if they are ill [27], which increases the possibility of misjudging the epidemic situation of infectious diseases just by calculating absenteeism.

Using artificial intelligence and information technology instead of manual methods to collect absenteeism data while considering the health information of students in attendance is highly important to solve the current development dilemma of SSS in China. To this end, an SSS was designed and trial-operated, which realized synchronous acquisition of identity, absenteeism, and temperature data based on face recognition and thermal imaging. The system has been in operation at 3 sentinel schools in the Yangtze River Delta region since November 2020. Data from these 3 schools collected by the system between March 2021 and January 2022 were exported to investigate 2 aspects. First, the alternative method of manually collecting absenteeism data explored in previous studies has produced a large amount of erroneous data [14]. Simultaneously, although infrared temperature measurement technology is widely used in the screening of suspected cases of infectious diseases in the population [28], instruments as well as environmental and individual factors can easily interfere with its accuracy [29-31]. Therefore, the completeness and accuracy of the collected data need to be verified. Second, body temperature is the first clinical symptom of most infectious diseases, and its advantage in influenza SSS has been confirmed by Miller et al [32]. Theoretically, multisource data can improve the accuracy of the perception of infectious disease information by the surveillance system [33]. The monitoring effectiveness of the combination of nonclinical symptoms (absenteeism) and clinical symptoms (fever) is another issue that requires investigation.

## Methods

### Reporting System

**Figure 1:**
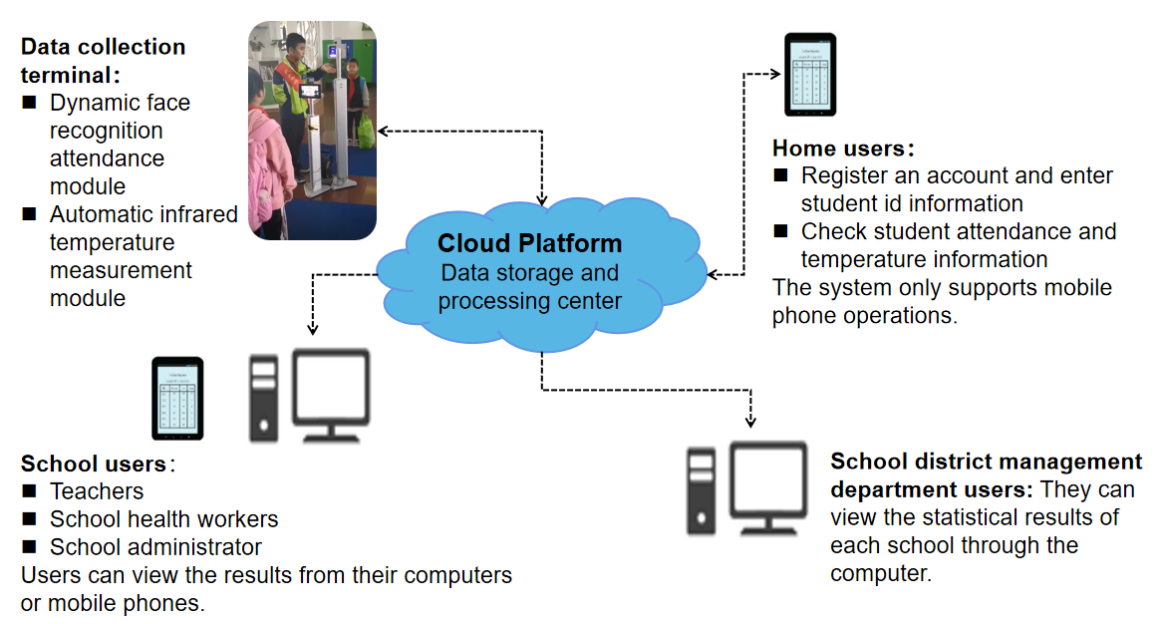
Workflow of the Xiao Lian Xing system.

The proposed SSS was based on an app called “Xiao Lian Xing” (XLX) [34], which can be downloaded for free on Alipay (Alibaba Group Holding Co, Ltd). Τhe app integrates face recognition technology and infrared temperature measurement technology to realize intelligent management of school public health. Its workflow is shown in Figure 1.

The operating company signed service agreements with the school and its superior management organization. According to the agreement, the school organized for the parents to register an account in Alipay with a smart phone (registration was completely voluntary), and then input information about the child, such as the name, gender, ID, school name, class, and face image into the account. Parents can also use the account to check the attendance and temperature of their children. The system collected student attendance and temperature data through terminal devices, which were composed of 2 modules, namely a face recognition system (identification accuracy ≥99.99%, Sunmi Technology Group Co, Ltd) and an infrared thermometer (measuring accuracy of ±0.5℃, Hikvision Digital Technology Co, Ltd). The 2 modules were connected using a self-designed software for synchronous input and upload of the identity, attendance, and temperature. The data processing center was responsible for processing the data uploaded by each terminal and relaying the analysis results of absenteeism and fever at different levels to family users (ie, parents or other guardians), school users (ie, teachers, school health workers, and administrators), and school district management department users.

**Figure 2:**
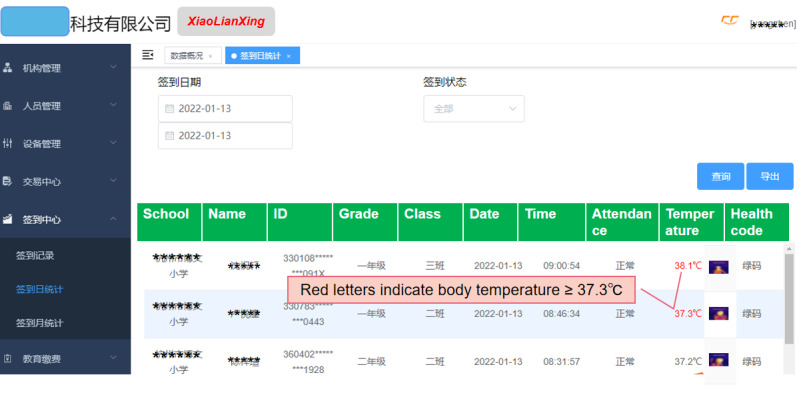
Statistical interface of student daily absenteeism and body temperature.

Terminal devices were generally arranged at the school gate. After students arrived at school every day, they went to the device for testing. Face recognition of the students was performed, and their identity information, attendance information, and facial temperature were automatically recorded by the instrument. Only students who were identified and had a normal temperature were admitted directly, whereas students with fever (temperature≥37.3℃) were processed separately. To ensure that every student was subjected to instrument testing, the school arranged for staff on duty to supervise the children. Daily information on student attendance and temperature was transmitted to the cloud data center in real time. The data processing center generated a daily statistical report of the check-in and body temperature (with infrared images) of students in real time (Figure 2), summarized and analyzed the data, and gave feedback on the results at different levels (individual, classes, schools, school districts, etc).

### Study Population and Data Collection

The study population comprised 3 schools in the Yangtze River Delta region. Schools A and B were primary schools. School A, located in Xiaoshan District, Hangzhou city, Zhejiang Province, began to use version 1.0 of the system (only face recognition) in November 2020 and version 2.0 (face recognition and infrared thermometer) in March 2021. School B, located in Binjiang District of Hangzhou city, began to pilot version 2.0 of the system in March 2021; it allowed students to use smart cards to check-in and began to fully use version 2.0 in September 2021. School C is a junior high school located in Pudong District, Shanghai, and it started to use version 2.0 of this system in October 2021.

The surveillance period was divided into 2 phases, namely, phase I, from March 1, 2021, to June 25, 2021, and phase II, from September 1, 2021, to January 14, 2022. The effective surveillance times of these phases were 83 and 91 days in total, respectively. In phase I, the numbers of students in school A and B were 1861 and 1100, respectively. In phase II, the numbers of students in schools A, B, and C were 1954, 1154, and 427, respectively, with a total of 3535 students. The system collected the following data for each school: total number of enrolled students, number of daily absentees, and number of daily students with fever. By default, students who failed to check-in within 1 hour after the specified arrival time were counted as absenteeism cases, where students whose body temperature was ≥37.3℃ were counted as fever cases.

The absenteeism reported by physicians in schools A and B (school C has no record of the school physician) was collected as the reference standard to evaluate the quality of absenteeism reported by the system. Absenteeism reported by the school physicians was defined as “the student is not in school that day.” The data reported by school physicians were collected from September 1, 2021, to January 14, 2022. To verify the reliability and feasibility of the surveillance system for infectious diseases, influenza was selected as the target disease. The reference criteria for influenza activities were obtained from the weekly influenza surveillance report released by Chinese National Influenza Center [35]. These weekly report statistics show the weekly positive rate of influenza virus (WPRIV) tests in southern and northern China. The WPRIV was calculated as the ratio of the number of virus-positive samples to the total number of samples submitted [36]. As both Hangzhou and Shanghai are in southern China, only the data of the southern region in the weekly report were used. The corresponding surveillance weeks ranged from the ninth week of 2021 to the fifth week of 2022. During this period, the influenza virus strain circulating in southern China was mainly type B.

### Ethics Approval

Data used in this study were anonymized, so the Tongji University Review Board classified this study as nonhuman subject research, and it was exempted from approval.

### Data Analysis

The daily absence rate (DAR) of the 3 schools was calculated as follows:



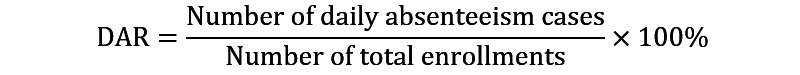



**Figure 3:**
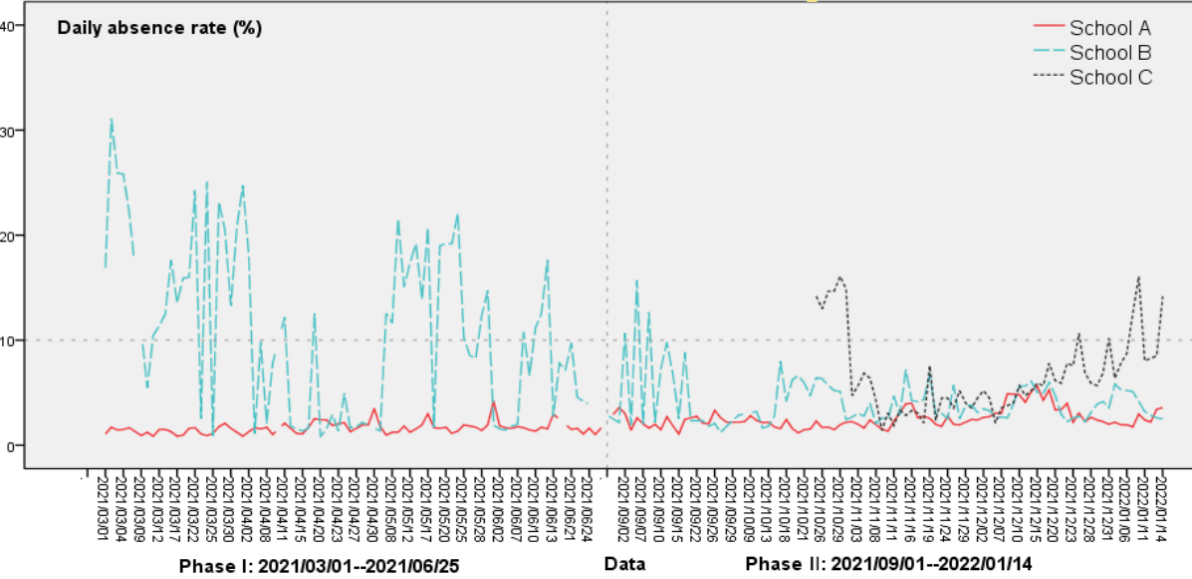
Time series of daily absenteeism rates reported by the systems of schools A, B, and C.

**Figure 4:**
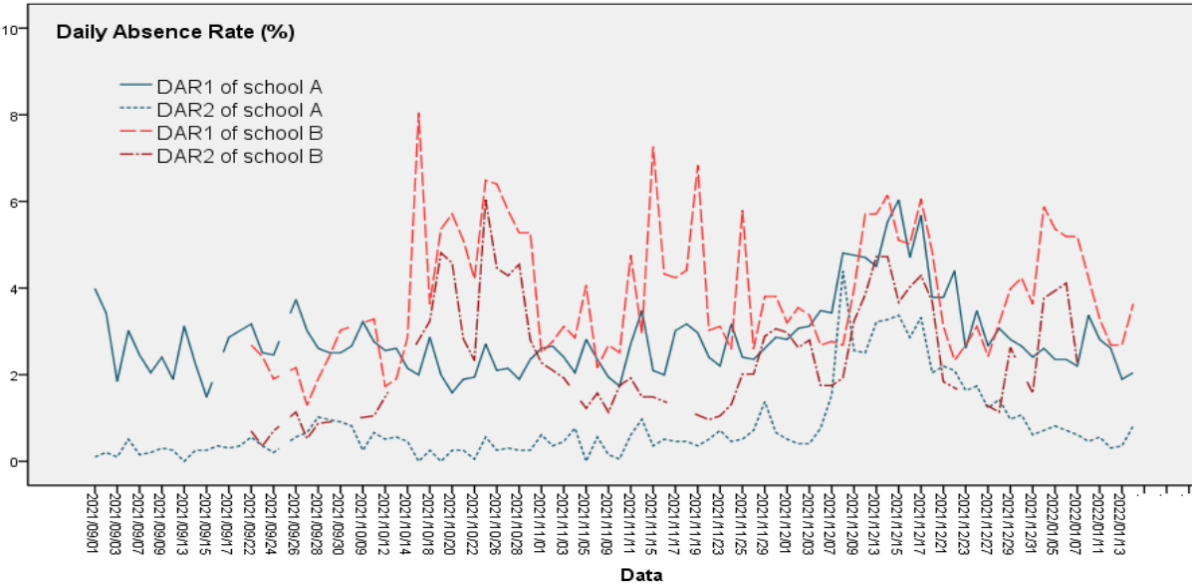
Time series of daily absenteeism rates reported by the system (DAR1) and school physicians (DAR2). DAR: daily absenteeism rate.

The DAR is divided into two categories: system-reported (DAR1) and school physician–reported (DAR2). The correlation coefficient of the 2 variables was then calculated. According to relevant literature [9,12], the data were considered abnormal if DAR1 exceeded 10%. The abnormal causes relating to the school were investigated, and if they were noninfectious factors, the abnormal data were statistically treated in an appropriate manner. Then, the DAR time series diagrams of the 3 schools were drawn (Figure 3), the Pearson correlation of the DARs among the 3 schools was calculated, and the morphological differences among their DAR curves were compared. We compared the correlation and trend of DAR1 and DAR2 (Figure 4).

Second, according to the time series diagram of DAR1 (Figure 3), the starting date was determined as the date when the DAR1 of each school began to stabilize, and then the daily fever rate (DFR) of each school was calculated after this date. When the DFR deviated by 3 SDs from the mean value, the infrared images of students in the system were examined. If the problem was operational, the corresponding DFR was used as the missing value and was replaced with the mean of the DFR for the previous and next days of that date. Absenteeism and fever were assumed to represent different severity degrees of influenza symptoms, with absenteeism representing severe symptoms and attendance with fever representing mild symptoms. Therefore, the denominators of both the DAR and DFR were set as the number of total enrollments. The calculation formula of the DFR was as follows:



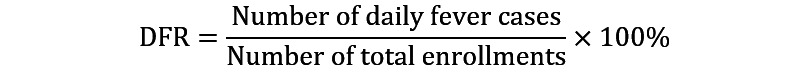



**Figure 5:**
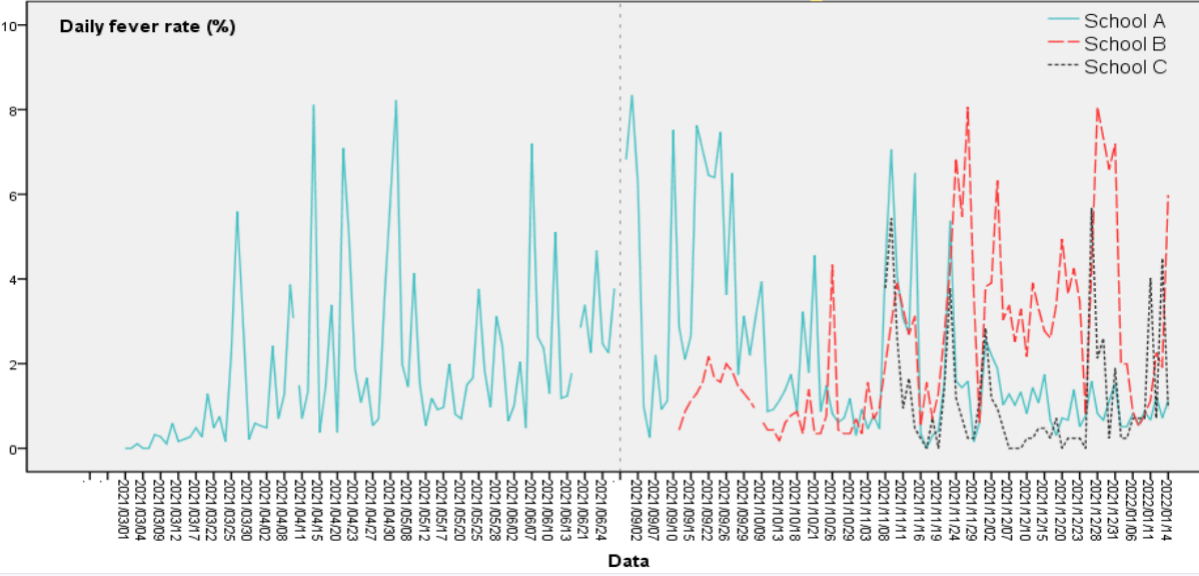
Time series of daily fever rates in 3 schools.

The DFR time series diagrams of the 3 schools are shown in Figure 5; the morphological differences among their DFR curves were compared, and the Pearson correlation of the DFRs among the 3 schools was calculated.

Third, based on the DAR and DFR, the weekly absenteeism rate (WAR) and weekly fever rate (WFR) for the three schools were calculated as follows:



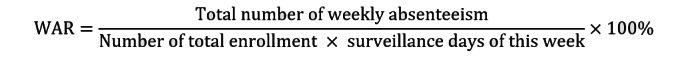





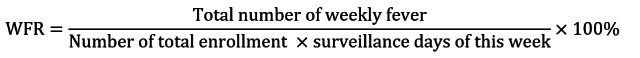



**Figure 6:**
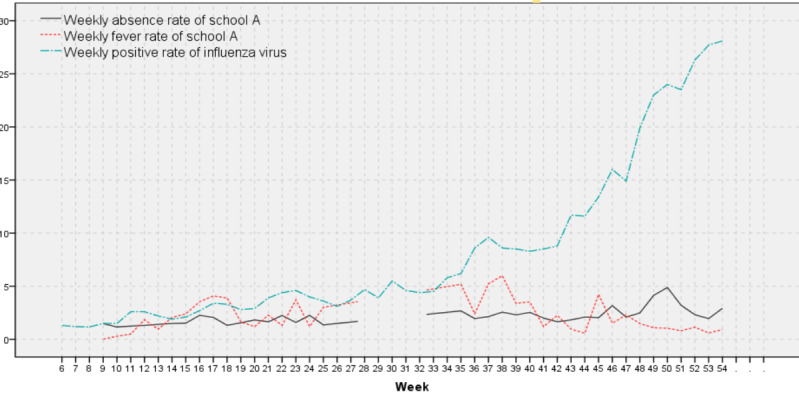
Weekly positive rates of influenza virus, weekly absenteeism rates, and weekly fever rates of school A.

**Figure 7:**
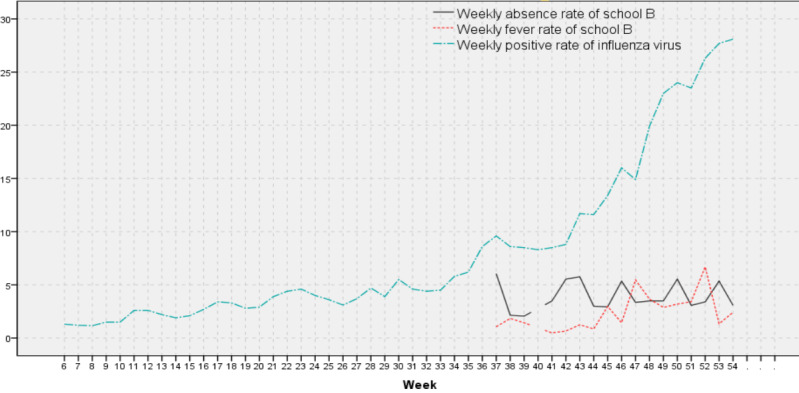
Weekly positive rates of influenza virus, weekly absenteeism rates, and weekly fever rates of school B.

**Figure 8:**
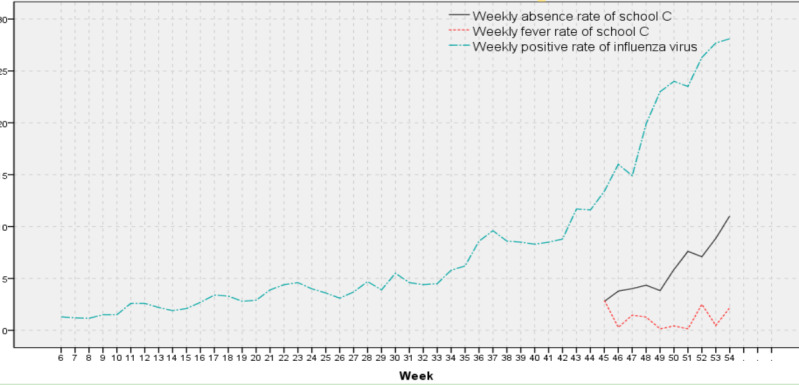
Weekly positive rates of influenza virus, weekly absenteeism rates, and weekly fever rates of school C.

The time series diagrams of the WAR and WFR of the 3 schools were produced, and their coincidence with the time series diagram trends of the WPRIV was compared (Figures 6-8).

Finally, the following statistical process was performed: (1) The sums of the WAR and WFR of each school were calculated. (2) Based on the data of school A, the data of school B were added at the beginning of week 37; then the data of school C were added again in week 45, and then the combined WAR and WFR were combined based on the weight of the total enrollment in each school. (3) The sums of the combined WAR and WFR were calculated. Simultaneously, the current time was set to *t*; then the WPRIV sequence was advanced by 1 week (*t– 1*), 2 weeks (*t–2*), and 3 weeks (*t–3*). The correlation of the WPRIV with the aforementioned WARs and WFRs and their sum was calculated under these 4 conditions to investigate the reliability, accuracy, and timeliness of different types of data derived from this system for influenza activity surveillance.

## Results

### Analysis of DAR1

In phase I, all 2961 students in schools A and B had registered accounts in the system. In phase II, the total number of students in the 3 schools was 3535, with 3530 students (99.86%) having registered accounts (Table 1). The DAR of school A remained relatively stable throughout the surveillance period, without surpassing the 10% critical threshold (Figure 3). The DAR of school B fluctuated sharply in phase I, but the overall trend was decreasing. In phase II, after about 2 weeks, the DAR curves of schools B and A were similar. The DAR of school C varied considerably during the first 2 weeks of system commissioning and began to stabilize about 2 weeks later. The DAR of school C exceeded 10% on 4 days, December 24, December 31, January 7, and January 10, 2022. These 4 days were weekends or holidays.

The DAR1s of the 3 schools were very similar along with the trends over the 30 monitoring days from November 8 to December 20 (Figure 3). During this time, 3 pairs of absenteeism rates, namely schools A and B (*r*_a_=0.508, *P*=.004), B and C (*r*_b_=0.427, *P*=.017), and A and C (*r*_ac_=0.447, *P*=.012), were moderately positively correlated. After December 20, the DARs at the 3 schools showed different trends, with the differences between school C and the other 2 schools being significantly different.

**Table 1:** Daily absenteeism rates and daily fever rates of 3 schools reported by the system in phases I and II.

School			Total number		Number of enrollments		Rate of enrollment (%)		Daily absenteeism rate (%)							Daily fever rate (%)				
									Minimum		Maximum		Mean		Minimum			Maximum		Mean
**Phase I**																				
	A	1861		1861		100		0.86		4.19		1.63		0			8.22		1.87	
	B	1100		1100		100		0.82		31.09		11.14		N/A^a^			N/A		N/A	
**Phase II**																				
	A	1954		1954		100		1.07		5.63		2.55		0			8.34		2.12	
	B	1154		1153		99.91		1.21		15.70		4.23		0.17			8.07		2.44	
	C	427		423		99.06		1.42		16.08		6.75		0			5.67		1.19	

^a^N/A: not applicable.

### Correlation Analysis of DAR1 and DAR2

Between September 1, 2021, and January 14, 2022, students from school A were expected to attend school for 92 days and students from school B for 90 days. Both the system and the school physician of school A recorded 92 days of data. A total of 5128 absences were reported by the system, whereas a total of 1476 absences were reported by the school physicians, with the latter accounting for 28.78% of the former. In school B, the system recorded 3452 absences over 90 days, whereas the school physician recorded 1691 absences over 66 days (73.3% completeness), with the number reported by school physicians being 48.99% of the number reported by the system.

A significant positive correlation between DAR1 and DAR2 in school A was detected (*r*=0.809, *P*<.001), and their change trends were highly consistent. In school B, DAR1 and DAR2 were also significantly positively correlated (*r*=0.766, *P*<.001), but there was a notable gap between them in November 2021 (Figure 4). The abovementioned data show that the absence reported by school physicians (completeness 86.7%) was only 36.5% of the absence reported by the system (completeness 100%), and that a significant positive correlation between the 2 was present (*r*=0.372, *P*=.002).

### Analysis of DFR

The DFR calculation time for school A started from March 1, 2021 (week 9), for school B from September 13, 2021 (week 37), and for school C from November 8, 2021 (week 45). The infrared image investigation of the system confirmed that high-temperature objects were placed next to the infrared thermometer in school A on 2 days (April 30, September 18), and on 1 day (April 1), the thermometer was unable to measure temperatures owing to physical failure. In school B, there were 2 days (November 11 and December 29) when a high-temperature object was placed next to the thermometer. The DFR of these 5 days was regarded as a missing value and replaced with the mean of the DFR for the previous and next days.

Overall, the DFR curve of school A resembles a parabola with a downward opening (Figure 5). This trend was similar to the daily average temperature change trend in this area during the surveillance period. During the 30 days from September 13 to October 29, the DFR of school A was always higher than that of school B, and after October 29, this relationship was gradually reversed. The DFR curves of schools C and A were closer, and the DFRs of these 2 schools were also significantly positively correlated (*r*_ac_=0.493, *P*<.001). Although no significant correlation was detected between schools A and B (*r*_ab_=–0.023, *P*=.84), and schools B and C (*r*_bc_=0.091, *P*=.54), the DFR curves of the 3 schools were consistent in some wave peaks and troughs.

### Correlation Analysis of WAR1, WFR, and WPRIV

The WAR1s and WFRs for the 3 schools were calculated, and their time series diagrams were plotted. The WAR1 of school A was consistent with the trend of the WPRIV (Figure 6), that is, both the WAR1 and WPRIV gradually increased over time. After week 42, influenza activity levels spiked, with the first peak of WAR1 of school A presenting in week 46 (1 week earlier than WPRIV), and the second peak in week 50 (the same time as the WPRIV). The first peak of the WFR1 in school A was at week 45 (2 weeks earlier than the WPRIV).

School B showed a peak of the WAR1 in weeks 42 and 43 (Figure 7). Investigation on this aspect showed that the school had problems with the health codes of some students; therefore, school B prudently allowed 4 classes of grade 1 to study at home. The second WAR1 peak of school B was in week 45, and the third one was in week 50. These 2 peak times were the same as those of school A, but school B had another WAR1 peak in week 53, which was different from school A. As the level of influenza activity increased, the WFR1 of school B peaked at week 45 (same as in school A), week 47, and week 52.

The surveillance weeks of school C were few, and the WAR1 of school C has been on the rise throughout the surveillance period (Figure 8), which was highly consistent with the trend of the WPRIV. The 3 peaks of the WFR1 in school C were in week 45 (same as school A and B), week 47, and week 52 (same as school B). On combining with Figures 5-7, the mismatch trend of the WAR1 and WFR became evident. This phenomenon was especially apparent in school B. For example, the WARs peaked in weeks 46, 50, and 53, whereas the corresponding WFRs were at a low point. For weeks 45, 47, and 52, the WFRs peaked, whereas the WAR1s were at their troughs.

Among the WAR1s reported by the system, only the WAR1 of schools A and C was significantly positively correlated with the WPRIV. At *t*–3, the correlation coefficient between the WAR1 of school A and WPRIV was the highest, whereas at *t*, the correlation coefficient between the WAR of school C and WPRIV was the highest (Table 2). Although the WAR of school B was not significantly correlated with the WPRIV in the 4 conditions, the variation trend of the correlation coefficients was similar to that in school A. Only the WFR of school B was significantly positively correlated with the WPRIV, and the maximum coefficient was at *t*–3. The addition of the WAR1 to the WFR produced an increased correlation with the WPRIV only in the sum of school B. The WAR1 of school A reported by the school physician was significantly positively correlated with the WPRIV, with the maximum coefficient located at *t*–3. The correlation coefficients between the WAR1 and WPRIV that were calculated based on system-reported data were higher than those calculated based on data reported by school physicians in each condition. The WAR1 of school B reported by both the school physician and the proposed system was not significantly correlated with the WPRIV in any of the 4 conditions.

**Table 2:** Correlation analysis of weekly absenteeism rate, weekly fever rate, and weekly positive rate of influenza virus.

Variables		Weekly positive rate of influenza virus							
		*t*	*P* value	*t–1*	*P* value	*t–2*	*P* value	*t–3*	*P* value
**Data reported by the system**									
	Weekly absence rate of school A	0.662	<*.*001	0.638	<*.*001	0.654	<*.*001	0.674	<*.*001
	Weekly absence rate of school B	0.334	*.*21	0.268	.32	0.391	.13	0.442	.09
	Weekly absence rate of school C	0.771	.009	0.728	.02	0.444	.2	0.222	.54
	Weekly fever rate of school A	–0.368	.03	–0.367	.03	–0.387	.02	–0.363	.03
	Weekly fever rate of school B	0.492	.05	0.589	.02	0.557	.03	0.618	.01
	Weekly fever rate of school C	0.229	.47	0.307	.33	0.056	.86	–0.002	>.99
	Weekly absence and fever rate of school A	0.058	.74	0.045	.79	0.038	.83	0.069	.69
	Weekly absence and fever rate of school B	0.6	.01	0.635	.008	0.687	.003	0.767	.001
	Weekly absence and fever rate of school C	0.686	.03	0.664	.04	0.317	.37	–0.333	.35
**Data reported by school physicians**									
	Weekly absence rate of school A	0.58	.007	0.55	.01	0.59	.006	0.648	.002
	Weekly absence rate of school B	0.429	.11	0.406	.13	0.4	.14	0.379	.16

## Discussion

### Principal Findings

In 2017, Groseclose and Buckeridge [37] proposed a surveillance system evaluation framework comprising 12 indicators, namely simplicity, acceptability, representativeness, stability, data quality, timeliness, flexibility, security, sensitivity, predictive value positive, cost-effectiveness, and standard use. Comparison of the proposed system with existing similar systems showed its advantages in in terms of simplicity, cost-effectiveness, data quality, sensitivity, and timeliness.

This system is simple to operate and has a low construction cost. The simplicity of the proposed surveillance system is reflected in four aspects: data availability and type, organization and type, data exchange and conversion, and personnel operation and training [37]. Sickness absence surveillance based on manual data collection requires the data reporter to have extensive medical expertise. Therefore, the reporter is required to be a school physician or a part-time worker with adequate medical training. On the other hand, the operation of the proposed system does not require professional personnel, but it only requires students to stand in front of the instrument in accordance with the standard for 1 second so that all symptom data can be automatically obtained. In this way, the system is highly cost-effective in terms of workforce. Moreover, contrary to the government-led SSS, which involves high investment for platform research and development, the proposed system is built based on the mature network platform and is thus cost-effective.

The absenteeism reported by the system was complete and accurate, and the completeness of the temperature was high, but the accuracy needs to be improved. Fingerprints, smart cards, or face recognition were used to guarantee that every student in attendance is checked and accurately identified. Lawpoolsri et al [14] attempted to use fingerprints instead of manual collection but failed after many students missed and checked wrongly. In this study, the proportion of students registering system accounts was close to 100%, the accuracy of face recognition technology was more than 99.99%, and the school also arranged personnel to supervise the students during check-in. Data showed that the absenteeism reported by the system was highly positively correlated with that reported by school physicians, and the completeness of the former was higher than that of the latter. The system also needs to confidently guarantee that the temperature of each identified student is accurately measured. As face recognition and infrared temperature measurement were almost synchronized, the completeness of temperature was adequate, but its accuracy needs to be improved. The accuracy of thermal imaging is affected by the instrument, environment, and individual factors [29-31]. In this study, only the temperature of school B met the monitoring requirements. The instruments in school B were installed indoors and strictly supervised by students. These measures are helpful to reduce improper measurement behavior and improve the accuracy. Future research will focus on the construction and installation of instruments and implementation of the operation standards.

The proposed system showed good sensitivity. The surveillance sensitivity includes case detection, outbreak detection, and case definition [37]. Existing systems focus on students who are absent from school [6-12,14-18,20-22], whereas others also consider students who visit the school health room [9,10]. During an influenza outbreak, about 20% of the population shows symptoms, but only about 2% require physician consultation [38]. Students do not miss school or consult school physicians unless they are seriously ill. Because of academic pressure, it is common for Chinese students to attend school sick, and about 74.7% of those who took sick leave were absent for less than 1 day [26]. This is a potential explanation for the number of absences reported by school physicians being only about 36.5% of the number reported by the system in this study. The correlation between the WAR of school A reported by the system and the WPRIV was higher than that between the WAR reported by school physicians and the WPRIV, thus appearing to support this conclusion. Data showed that in the influenza outbreak season, the DFR in some schools was up to 8%. By adjusting the absenteeism criteria and combining temperature screening, the presented system is more sensitive to case and outbreak detection than existing systems.

The results also showed good timeliness rates. The data of this system were transmitted in real time and analyzed automatically. Parents, teachers, and administrators were able to instantly access the attendance and temperature status of an individual, class, and school. Baer et al [11] proposed that the surveillance value of absenteeism lies more in situational awareness than in early detection, which may need to be revised when applied to China. This study revealed that absenteeism in the 3 schools was significantly positively correlated when influenza activity levels were low, which was not consistent with the findings of Schmidt et al [8] stating that absenteeism is very similar across age groups. With the increase in influenza prevalence, absenteeism in primary schools was the first to peak, and absenteeism in middle schools reached the first peak 2 weeks later, which is consistent with the findings of Mook et al [39]. Subsequently, the gap in the absenteeism timing among schools increased, even between 2 primary schools; at this point, the claim of Schmidt et al [8] does apply. The addition of temperature data increased the timeliness of the system. The results showed that temperature could predict influenza up to 3 weeks earlier when the temperatures of students were accurately measured. Combined with the study of Miller et al [32], the timeliness advantage of temperature in influenza surveillance may indeed exist. Therefore, it is extremely critical to standardize instrument placement and operation procedures in the future to further improve the reliability and accuracy of temperature data.

### Limitations

First, although thermal infrared imaging is simple, its accuracy is easily influenced by environmental factors [29]. Because of the need for COVID-19 prevention, some schools introduced the proposed system. Nonetheless, these schools attach different importance levels to epidemic prevention and control, which in turn leads to different levels of hardware support and operation supervision. These disparities led to differences in data quality, especially temperature data, which hindered further analysis of these data. Second, COVID-19 was a prevailing issue during the surveillance period. Although it facilitated the roll-out of the system to a certain extent, the number of COVID-19 cases in the surveillance area was too small to be the target disease. However, because influenza was selected as the target infection, the status data for the influenza epidemic in general are distorted by the pressure of COVID-19 prevention. Consequently, some of the presented results need to be re-evaluated without considering the impact of COVID-19. Finally, although the system has been authorized by the government to collect sensitive personal information, possible changes to the national personal information and privacy protection policy in the future will substantially affect the operational stability of the system.

### Conclusions

Comparison of the proposed system with existing similar systems showed its advantages in terms of simplicity, cost-effectiveness, data quality, sensitivity, and timeliness. This study showed that the absenteeism recorded using face recognition technology was reliable, but the accuracy of the temperature recorded by infrared thermometers should be enhanced. The implementation of the influenza SSS based on absenteeism and temperature data was feasible. When influenza activity levels were moderate, a significant positive correlation between the DARs was detected; however, as the levels increased, the gap among the DARs gradually increased and peaked about 2 weeks earlier in primary schools than in junior high schools. The introduction of temperature measurement substantially strengthened the surveillance timeliness, allowing detection of influenza outbreaks up to 3 weeks earlier than traditional systems. This study demonstrated a feasible way to solve the challenge of developing a surveillance system and promote the automation of symptom data acquisition in the surveillance system.

## Abbreviations

DAR: Daily absence rate
DFR: Daily fever rate
SSS: Syndromic surveillance system
WAR: Weekly absence rate
WFR: Weekly fever rate
WPRIV: Weekly positive rate of influenza virus
